# Transformational Role of Medical Imaging in (Radiation) Oncology

**DOI:** 10.3390/cancers13112557

**Published:** 2021-05-23

**Authors:** Catherine Coolens, Matt N. Gwilliam, Paula Alcaide-Leon, Isabella Maria de Freitas Faria, Fabio Ynoe de Moraes

**Affiliations:** 1 Department of Medical Physics, Princess Margaret Cancer Centre & University Health Network, Toronto, ON M5G 1Z5, Canada; matthew.gwilliam@uhn.ca; 2 Department of Radiation Oncology, University of Toronto, Toronto, ON M5T 1P5, Canada; 3 Department of Medical Biophysics, University of Toronto, Toronto, ON M5G 1L7, Canada; 4 Department of Biomedical Engineering, University of Toronto, Toronto, ON M5S 3G9, Canada; 5 TECHNA Institute, University Health Network, Toronto, ON M5G 1Z5, Canada; 6 Joint Department of Medical Imaging, University Health Network, Toronto, ON M5G 1Z5, Canada; paula.alcaideleon@uhn.ca; 7 Harvard Program in Global Surgery and Social Change, Harvard Medical School, Boston, MA 02115, USA; bellinhamariah@ufmg.br; 8 Department of Oncology, Division of Radiation Oncology, Queen’s University, Kingston, ON K7L 5P9, Canada; Fabio.YnoedeMoraes@kingstonhsc.ca

**Keywords:** imaging biomarker, microenvironment, brain cancer, quantitative imaging, radiation oncology, response, RECIST

## Abstract

**Simple Summary:**

Onboard, imaging techniques have brought about a huge transformation in the ability to deliver targeted radiation therapies. Each generation of these technologies enables us to better visualize where to deliver lethal doses of radiation and thus allows the shrinking of necessary geometric margins leading to reduced toxicities. Alongside improvements in treatment delivery, advances in medical imaging have also allowed us to better define the volumes we wish to target. The development of imaging techniques that can capture aspects of the tumor’s biology before, during and after therapy is transforming how treatment can be delivered. Technological changes have further made these biological imaging techniques available in real-time providing the opportunity to monitor a patient’s response to treatment closely and often before any volume changes are visible on conventional radiological images. Here we discuss the development of robust quantitative imaging biomarkers and how they can personalize therapy towards meaningful clinical endpoints.

**Abstract:**

Onboard, real-time, imaging techniques, from the original megavoltage planar imaging devices, to the emerging combined MRI-Linear Accelerators, have brought a huge transformation in the ability to deliver targeted radiation therapies. Each generation of these technologies enables lethal doses of radiation to be delivered to target volumes with progressively more accuracy and thus allows shrinking of necessary geometric margins, leading to reduced toxicities. Alongside these improvements in treatment delivery, advances in medical imaging, e.g., PET, and MRI, have also allowed target volumes themselves to be better defined. The development of functional and molecular imaging is now driving a conceptually larger step transformation to both better understand the cancer target and disease to be treated, as well as how tumors respond to treatment. A biological description of the tumor microenvironment is now accepted as an essential component of how to personalize and adapt treatment. This applies not only to radiation oncology but extends widely in cancer management from surgical oncology planning and interventional radiology, to evaluation of targeted drug delivery efficacy in medical oncology/immunotherapy. Here, we will discuss the role and requirements of functional and metabolic imaging techniques in the context of brain tumors and metastases to reliably provide multi-parametric imaging biomarkers of the tumor microenvironment.

## 1. Introduction

Since on-board cone-beam computed tomography (CBCT) systems entered the market in 2005, their use has enabled image-guided radiotherapy to become the routine standard of care for the majority of radiotherapy procedures in the second decade of the century. This has resulted in improved tumor control and reduced toxicities for patients [1]. Since then, the radiotherapy landscape has seen multiple paradigms evolve, driven by imaging [2,3]. Of these, quality, toxicity reduction, dose escalation, and hypo-fractionation, largely relate to the accuracy and precision of delivery which, while not yet fully exploited, are well described in the literature and are being developed in clinic through the use of systems such as linear accelerators with onboard MRI and even the prospect of MR-guided particle therapy [4,5].

The other paradigms, voxelization and adaptation, and to it we would add ‘response’, are the focus of this paper. Their evolution is reliant on the functional and metabolic aspects of imaging which are yet to be fully exploited but could significantly enhance disease outcomes [6]. Their use would allow the tissue microenvironment to be probed, both on a whole tumor and voxelwise basis. This in turn would enable techniques such as dose painting during radiotherapy planning; treatment adaptation; as well as an assessment of treatment response and tumor micro-environmental changes in a more biologically relevant way. Early intervention and adaptation are essential in the management of cancer therapy and key components of the personalization of care.

There exists a large range of imaging techniques, from the more “direct” signal interrogation of tumor physiology using computed tomography (CT) and magnetic resonance imaging (MRI) with or without the use of contrast-enhancing tracers; to more “indirect” approaches often referred to as “metabolic” imaging where the imaging signal is a by-product of tumor metabolism, often measured with positron emission tomography (PET) following either glucose or oxygen consumption mechanisms. Here, we will take a closer look at the role of various functional imaging techniques in the context of brain tumors and metastases treated with both stereotactic radiosurgery and (systemic) immunotherapy. Using experience with Phase 1/2 trials, we will discuss the feasibility of obtaining functional parametric images, how they can describe various components of the tumor microenvironment and how they can reliably be used to both plan and evaluate treatment response. This will start with an in-depth look at the concept of what constitutes a reliable clinical endpoint. Biological imaging will focus mostly on flow-based and metabolic MRI and PET techniques. Reviewed works were chosen to support the hypotheses and potential transformative practices of a biological personalization to cancer treatment rather than providing a full systematic literature review. The reliability of using multiparametric techniques in routine radiation oncology practices will be discussed as well as the relevance of computational image processing techniques and use of artificial intelligence. It is now known that metabolic reprogramming in brain tumors is influenced by the tumor microenvironment, hence contributing to drug resistance and tumor recurrence. Many of these aspects of altered metabolism provide novel therapeutic opportunities to effectively treat primary brain tumors, but furthermore, altered cancer metabolism can be leveraged to noninvasively image tumors, which facilitates improved diagnosis and the evaluation of treatment effectiveness. 

## 2. Evaluation of Treatment Response

Defining reliable endpoints is an essential part of treatment assessment, and still presents as a challenge to the oncology community. Progression-free-survival (PFS) has become increasingly important and with it, the ability to radiologically assess progression [7]. Although response rate, overall survival (OS), and PFS are well-known indicators of treatment efficacy, several other aspects have been studied to assess responses associated with clinical benefits to the patient, ranging from quality of life to the need of further treatments [8]. Endpoints in oncology can be objective or subjective, the first ones being preferred due to reducing self-report biases and being more accurate when reported [9]. Objective or standardized tests are usually based on function (e.g., objective cognitive testing), while subjective tests rely usually on symptoms and signs and their burden to the patient (patient-reported outcome). Some current endpoints being evaluated in neuro-oncology are cognitive standardized testing, symptom burden, quality of life, seizure activity and corticosteroids use. 

Identification and delineation of tumor burden on imaging is a critical, yet challenging, function for neuro-oncology practice. Currently, the gold standards for the radiological assessment of treatment progression/response are the Response Evaluation Criteria in Solid Tumors (RECIST) [10] or the Radiological Assessment of Neuro-Oncology Criteria (RANO) [8]—the latter being more oriented towards assessment with MRI of the brain. Both of these techniques characterize disease as either stable, progressing, partially responding, or completely responding, based on changes in the dimensions of the tumor observed on radiological imaging. Anatomical changes in the tumor burden on different imaging sequences are used as surrogate for efficacy throughout the entire treatment journey.

One of the main weaknesses of these anatomical methods is the lack of any functional/biological dimension in the assessment. This was partially acknowledged in the development of the original RANO which, explicitly excludes tumors exhibiting greater enhancement after radiation treatment. These weaknesses have been exacerbated in recent years as the use of immunotherapy has grown, which can induce an immune response around the tumor, shown as enhancement on imaging, which can appear radiologically as progression—the extreme scenario being an immunotherapy-responding patient being incorrectly moved to palliative management based on inaccurate response assessment. Pseudo-progression is a confounder to both RECIST and RANO. In response, iRECIST [11] and iRANO [12] have been developed, which concede that the decision on treatment response must be delayed and confirmed with later images when dealing with patients undergoing immunotherapy. Hence, there is still a need to develop methods that distinguish treatment failure from pseudoprogression. 

The RANO efforts have also advocated for the use of clinical status as an endpoint, to assess response to treatment of different types of brain tumors. One example is leptomeningeal metastases (LM), a feared complication that can arise with tumor progression, usually presenting with discordant clinical and radiological features. Protocols such as the RANO-LM have been developed to refine recommendations of endpoints and to response criteria to specific settings, adding domains such as gait, strength, level of consciousness, and behavior to the Neurological Assessment in Neuro-Oncology (NANO) assessments [8,13]. However, these criteria still need validation and are restricted to clinical trials. Nowadays in neuro-oncology, disease progression is mostly assessed and defined using qualitative and quantitative evaluation of tumor burden (tumor is measured using two diameters) before, during, and after therapy. However, they are many other variables correlated to clinically meaningful progression including quality of life and or neuro-cognition [14]. 

Quality of life (QoL) and neuro-cognition have been in the spotlight recently for becoming an essential endpoint to be established, especially since patients are experiencing longer survival rates because of more effective treatment. It is imperative to consider QoL as clinical trial endpoints because it not only directly impacts the patients’ everyday lives, but it has been shown that morbidity correlates to poor prognosis, for example, the worsening of cognitive function [8]. Objective QoL and neurocognitive assessments are ultimately preferred since they increase reproducibility and decrease self-report bias. 

Additionally, accurate criteria regarding optimal timing of post-treatment tumor imaging still poses a challenge to radiologists. That means, finding the balance between assessing imaging early enough to catch an incomplete response and proceed with new intervention; and allowing enough time for complete tumor response and the resolution of radiotherapy-induced inflammation. Because in many tumors types appropriate timing of imaging is still not a consensus, many protocols such as RANO, i-RANO, i-RECIST and RANO-LM are raising efforts to establish reliable endpoints and enable widespread clinical adoption of the new therapies. Future work should apply the present model to new and functional/quantitative imaging technique, in addition to leveraging the power of artificial intelligence. 

## 3. Biological Imaging

Medical image biomarkers of cancer promise improvements in patient care through advances in precision medicine. Compared to genomic biomarkers, image biomarkers provide the advantages of being non-invasive, and characterizing a spatially heterogeneous tumor in its entirety, as opposed to limited tissue available via biopsy. Additionally, the understanding that functional and quantitative imaging of the tumor microenvironment is better poised to capture changes in tumor physiology and functional behavior following treatment much sooner than radiological measurements spurred the exponential effort in imaging biomarker development. A key component in capturing biological changes is understanding tumor heterogeneity, hence the importance of voxelization. Tumor heterogeneity is due, at least in part, to the stability of multiple genomic clonal populations within a neoplasm [15]. These arise from divergent evolution of the originating cells’ progeny and may be sustained by geographical isolation within the tumor and cooperation between clones. Tumor-extrinsic features of the extracellular matrix (ECM) and tumor microenvironment (TME) lead to dynamic interplay between epigenetically regulated phenotypes. Spatial heterogeneity in TME components across a tumor may reinforce intra-tumor heterogeneity, whereas global changes in TME between patients influences inter-tumor heterogeneity [16].

### 3.1. Flow-Based Physiological Imaging

One aspect of the TME that has been heavily investigated is tumor angiogenesis and methods focusing on (indirect) metrics of the permeable nature of tumor microvasculature. Dynamic Susceptibility Contrast-enhanced (DSC) and Dynamic Contrast-Enhanced (DCE) are MRI perfusion techniques that use an intravascular, non-diffusible, exogenous Gadolinium-based contrast agent. DSC emphasizes the susceptibility effects of Gadolinium-based contrast agents on the signal echo, using a series of T2- or T2*-weighted images. DCE exploits the relaxivity effect of Gadolinium-based contrast agent on the signal echo, acquiring serial T1-weighted images before, during and after its administration [17,18].

#### 3.1.1. Dynamic Susceptibility Contrast (DSC) Imaging

**Figure 1:**
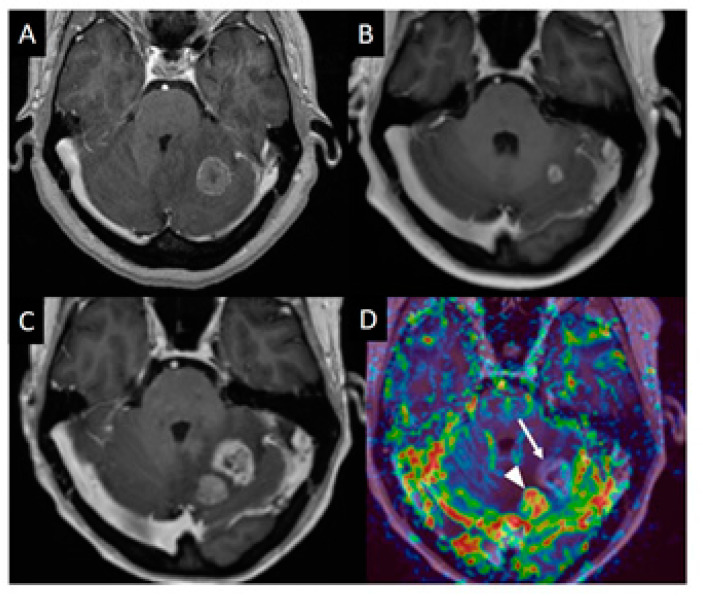
T1-weighted and DSC imaging of a left cerebellar metastasis. (**A**) Post contrast T1-Weighted MRI image of a 40 yo (year old) female with colon cancer and left cerebellar metastasis. (**B**) MRI 7 months later showed marked reduction is size after radiosurgery treatment. (**C**) MRI performed 11 months after treatment showed increase in size of the treated lesion and a new enhancing lesion medial to it. (**D**) CBV map from DSC perfusion (arrow) demonstrates mainly low perfusion. Some small foci of slightly elevated perfusion may represent foci of recurrent tumor on a background of predominant radiation necrosis.

DSC is the most commonly used MR perfusion technique. It is acquired during the first pass of a compact gadolinium bolus through the brain vessels and generally employs an echo planar T2*-weighted gradient echo sequence with a temporal resolution of 1–2 s. Total acquisition time is usually less than 2 min. Gadolinium shortens the T2 and T2* of the tissue resulting in decreased signal along the vessels and tissues surrounding the vessels. Multiple parameters can be derived from this technique, with cerebral blood volume (CBV)—which reflects vessel density—being the most frequently used in brain tumors (see Figure 1). DSC does not provide absolute CBV values, therefore values are often expressed as relative metrics compared to normal appearing white matter or grey matter (rCBV). A new DSC technique consisting in the simultaneous acquisition of gradient echo and spin echo DSC perfusion shows great promise [19]. This technique provides, not only an estimation of vessel density but also an estimation of mean vessel size and vessel type (arterial or venous dominance).

DSC perfusion is used in clinical practice for characterization of brain masses, glioma grading, and, most commonly, for the differentiation between treatment-related abnormalities mimicking tumor progression and true recurrent tumor. Many studies have investigated the use of DSC derived CBV for differentiation between treatment related changes and true tumor progression in gliomas and brain metastasis [20,21,22,23]. The two most common approaches to measure CBV are mean lesion value and maximum lesion value (“hot spot” approach). A recent meta-analysis showed pooled sensitivities and specificities for detecting tumor recurrence in high-grade gliomas using the mean rCBV (threshold range, 0.9–2.15) and maximum rCBV (threshold range, 1.49–3.1) corresponding to 88% and 88% (95% CI: 0.81–0.94; 0.78–0.95) and 93% and 76% (95% CI: 0.86–0.98; 0.66–0.85), respectively [20]. In a head-to-head comparison between DCE and DSC for the differentiation between tumor recurrence and radiation necrosis in treated high grade gliomas, DSC perfusion showed higher diagnostic accuracy than DCE perfusion [24]. 

DSC relies on the dynamic susceptibility induced by the first pass of a gadolinium bolus through the brain vessels. The presence of blood, calcium or air-bone interfaces can result in background susceptibility artefact that severely impairs our ability to measure perfusion. In particular, the presence of hemorrhage, very often precludes perfusion evaluation in hemorrhagic brain metastasis and high-grade gliomas. In order to achieve high temporal resolution, the spatial resolution and signal to noise of DSC perfusion are lower than in DCE, which limits the evaluation of small lesions. In addition to these technical limitations, similar to DCE, DSC suffers high variability in the optimal reported thresholds [20]. 

#### 3.1.2. Dynamic Contrast Enhanced (DCE) Imaging

DCE is a technique that investigates microvascular structure and function by tracking the pharmacokinetics of the injected contrast medium as it passes through the tumor vasculature. This technique is used in both CT as well as MR imaging using low molecular weight Iodine- and Gadolinium-based contrast agents respectively [25,26,27,28]. The increased tumor signal intensity measured following injection—referred to as a time attenuation curve (TAC)—reflects its perfusion, vascular permeability and extracellular volume. The combination of pharmacokinetic modeling with TACs is sometimes referred to as ‘perfusion’ imaging, which allows for the derivation of quantitative and semi-quantitative metrics. Models vary from simple first-pass uptake derivations, to compartmental and distributed parameter models [29,30]. Some of the most widely utilized metrics are the volume transfer coefficient (K_trans_) of contrast between the blood plasma and the extracellular extra-vascular space (Ve) derived from the 2-compartmental Tofts model, which aims to represent the permeability of the tumor vasculature. The latter has been shown to be higher in tumor recurrence than in radiation necrosis [31,32]. A more recent study by Chen et al. (2018) evaluated multi-parametric MRI as a biomarker for NCS-specific progression-free survival (PFS) and overall survival (OS) in brain metastases patients from primary breast cancer. They found in multivariate analyses, that Δ K_trans_ and ΔPeak were independent prognostic factors for CNS-specific PFS and OS, respectively, after controlling for age, size, hormone receptors, and performance status [33]. 

The importance of radiation dose was investigated in another study by Winter et al. (2018) where they compared the direct voxel-wise relationship between dose and early MR biomarker changes both within and in the high-dose region surrounding brain metastases following stereotactic radiosurgery (SRS). Longitudinal changes were investigated by computing absolute ΔADC, Δ K_trans_ and ΔVe for day 3 and 20 post-SRS relative to day 0. Only Ve exhibited significant differences between day 0 and 20 and day 3 and 20 within the gross tumor volume (GTV) following SRS. Strongest dose correlations were observed for ADC within the GTV and weak correlations were observed for ADC and K_trans_ in the surrounding >12 Gy region most likely reflecting underlying vascular responses to radiation [34].

Although the quantitative and semi-quantitative analyses are of great interest in scientific investigation, the additional benefit in clinical routine is still controversial. The reasons for this are varied but can likely be grouped into tumor heterogeneity and the need for voxel wise analysis on the one hand and the lack of validation and standardization in the analysis methods on the other hand [3,35]. The requirement for voxel wise analysis is clearly demonstrated in a study developing a parametric temporal approach to voxel-based tissue classification and analysis from 4D DCE CT of the brain [36]. It is was shown that the voxel-wise approach is more reproducible and sensitive compared to conventional 2D DCE analysis and resulted in greater accuracy and reliability in measuring changes in perfusion CT-based kinetic metrics in patients with metastatic brain cancer treated with SRS.

**Figure 2:**
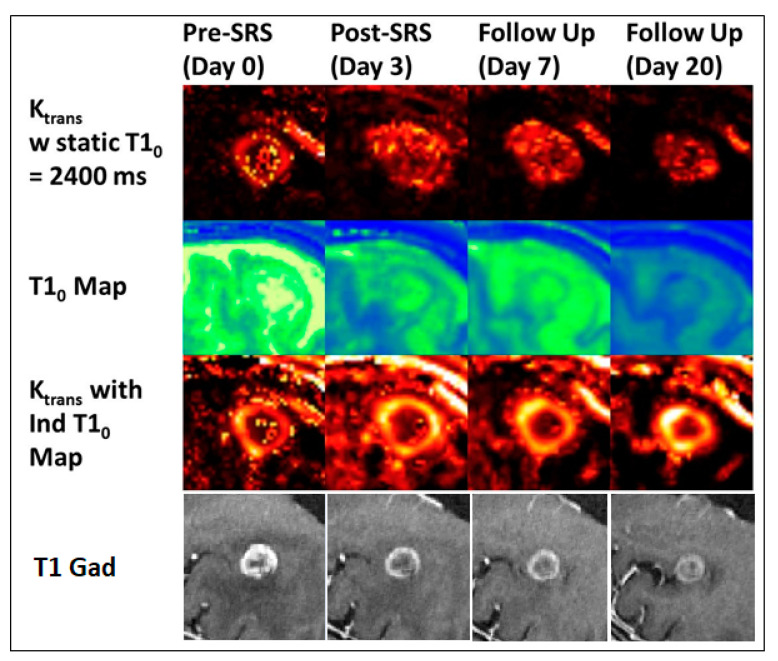
Central section through a brain metastasis for the same patient over different imaging days showing (**top**) K_trans_ values using a static T10 map, (**middle**) the voxel-based T10 map, and (**bottom**) K_trans_ values using the individual T10 map [37].

Utilizing the phantom-validated DCE CT parametric analysis as a gold standard in terms of contrast enhancement, a follow-up study then set out to compare the early detection of tumor response using DCE MRI against 4D DCE CT in the same patient cohort [35,36]. Perfusion images were acquired in the same patient on the same days with both modalities over the course of treatment. In a direct (co-registered) voxel-to-voxel comparison, Pearson analysis showed statistically significant correlations between CT and MR perfusion which peaked at day 7 for K_trans_ (R = 0.74, P < 0.0001) (see Figure 2). The strongest correlation to DCE-CT measurements however was found with DCE-MRI analysis using voxel-wise T10 maps instead of assigning a fixed T10 value [37], highlighting the need for voxelization to capture an accurate representation of tumor heterogeneity. Comparison of histogram features also showed statistically significant correlations between modalities over all tumors compared to average signal values, confirming the importance of voxelwise approaches. Despite the differences in contrast agents, these are some of the highest multi-modal correlations reported in the literature and this is attributed to the detailed patient setup and image registration but equally, the standardized analysis method used in both techniques following conversion of TAC to contrast enhancement curves [38]. 

A multi-institutional study in H&N cancer investigated the variability in quantitative metrics from DCE MRI with different algorithm implementations of the Tofts family of models [39]. The results highlight the need for cross-algorithm quality assurance to enable interpretable DCE MRI results but showed comforting results when different institutions used a similar software.

Some of the hesitancy in clinical translation of perfusion-based pharmacokinetic metrics is rooted in the limitations and variability of existing modeling approaches. A return to semi-quantitative and qualitative parameters seems perhaps more practical, and therefore potentially more robust, in evaluating treatment but will not provide any personalization of care nor fully utilize the potential that biological parameters can provide if properly validated. 

Validation of DCE perfusion techniques continues to be a topic of interest with the RSNA’s Quantitative Imaging Biomarker Alliance (QIBA) DCE Profile whose most recent claim supports that a measured change in K_trans_ of a brain lesion (glioblastoma multiforme, GBM) of 21% or larger indicates that a true change has occurred with 95% confidence [40]. Part of the reason for these relatively large thresholds of detectable change are the lack of test-retest data available in the literature as will be discussed later. Another reason, however, is in large due to the limitations of commonly used compartmental models to capture the underlying tumor permeability and perfusion on a voxel wise basis. Their simplicity encouraged their wide use in oncological research, but they do not account for the roles of diffusion and convection in driving tracer transport. At the level of a voxel, this translates to the assumption of no cross-voxel tracer exchange, often leading to the misinterpretation of derived perfusion parameters [17,18,19,20]. Newer, more advanced transport models have since been proposed. Sinno et al. devised a Cross-Voxel Exchange Model (CVXM) that describes the exchange of tracer between the vasculature and the tissue, as well the transport of the molecules through the extravascular space (governed by diffusion and convection) [41]. CVXM’s advantages over existing models [8,18,19,20,22] are (1) its consideration of cross-voxel exchange at the voxel level, thus maintaining conservation of mass; and (2) its incorporation of all three transport processes allowing, when paired with DCE-MRI, a comprehensive measurement of kinetic parameters in all of the tumor’s interstitium: i.e., periphery and center.

#### 3.1.3. Arterial Spin Labeling

Arterial spin labelling uses the magnetic labelling of protons in blood to create an endogenous contrast agent. For example, blood excited, or ‘tagged’ in the carotid artery will, after a period of a few seconds, arrive in a tumour in the brain and change the signal obtained from an image taken of the tumour at that moment. By subtracting an obtained without the tagged blood from an image with the tagged blood creates an image of cerebral blood flow. The quantitative nature of this technique makes it particularly appealing non-invasive alternative to metrics obtained from DCE and DSC techniques. 

Various studies have been conducted to evaluate the role of ASL derived tumour blood flow for both tumour grading/differentiation and treatment response with some promising results [42,43,44,45,46]. Some authors have suggested that ASL may be more sensitive in distinguishing tumour recurrence and pseudoprogression than other, more invasive techniques [44,47]. However, this promising modality has yet to enter more commonplace clinical practice. Factors such as limited signal to noise have largely limited the technique to large research settings with high field strength magnets.

### 3.2. Metabolic Imaging

#### 3.2.1. Diffusion Weighted Imaging (DWI) 

Diffusion-weighted-images provide unique contrast and are used clinically to distinguish benign from malignant tumors, identify recurrent tumors, and monitor treatment response [48]. Diffusion-weighted-imaging-(DWI) is an MRI technique sensitive to the underlying random movement of water molecules in the tissue such as diffusion. Its origins date back to the 1960s with Stejkal and Tanner [49] whose sequences and mathematical descriptions still broadly define the technique. The reader is directed to many excellent reviews for a fuller description of the physics [50,51,52,53,54] but in its simplest Stejkal-Tanner form, two identical gradients are applied either side of the 180° pulse in a spin-echo sequence. The 180° pulse means that stationary spins brought out of phase by the first gradient will be brought back into phase by the second gradient—i.e., the resulting echo will be the same as it would be without the two gradients. However, if the spins move in space over the course of the experiment, the gradients they experience will not be equivalent and opposite resulting in the spins being, at least partially, out of phase when the echo is produced—the signal will be attenuated. Depending on the implementation, the ‘b-value’ of a DWI sequence is a product of, amongst other variables, the gradient strength (mT) and gradient duration (s). Repeating the sequence with increasing ‘b-values’, usually by increasing the gradient strength, exacerbates the signal attenuation due to the random movement of water molecules. 

Signal loss in DWI can be used to generate two important biomarkers: Intra-voxel Incoherent Motion (IVIM), related to tissue perfusion, and Apparent Diffusion Coefficient (ADC), related to tissue cellularity. IVIM signal loss is partly due to water molecules flowing in the pseudo-random geometry of capillaries moving over the course of DWI sequence. Tissues that have a greater density of capillaries, i.e., more perfused tissues, will have greater IVIM signal loss. Diffusion related signal loss is due to water molecules moving at around 1/10th of the speed. As the diffusion of water molecules in tissue is influenced by the density of cell membranes, signal loss due to diffusion is a function of the tissue structure and is characterized by ADC. 

To separate signal losses due to ADC and IVIM, the speed of the water molecules in the different processes is exploited. At low b-values (<~250 ms) [55], the gradient strength gradient-duration product is too small to see large signal losses due to diffusion i.e., water molecules will not, in general, diffuse far enough, fast enough, between the two identical but opposite gradients to experience significantly different field strengths and subsequent signal loss. However, water molecules in the capillaries moving approximately 10 times faster will experience very different field strengths. Hence, at low b-values, observed signal loss is dominated by water molecules in the capillary bed. Therefore, IVIM signal loss can be quantified using low b-value sequences. At higher b-values, signal loss occurs mono-exponentially with increasing b-values due to diffusion. Diffusion related signal loss within a voxel can be modelled and an apparent-diffusion-coefficient (ADC) image produced—a biomarker of the tissue’s cellular structure [56].

IVIM has been described since the early days of DWI [57], but has enjoyed a renaissance in recent years, particularly in oncology, where need for vascular biomarker has been a focus. Correlation between IVIM and perfusion metrics produced by DCE-MRI [58] have been shown in some studies with IVIM having the obvious advantage of needing no contrast agents with their associated risks. However, other physiological processes such as glandular secretion and tubular flow compromise the perfusion-only model of IVIM [59,60].

ADC is non-specific and may be indicative of a range of underlying physiological changes such as necrosis, vascular development, or tumor cell proliferation. However, there is much literature correlating changes in ADC with outcomes in various types of tumor including in glioma, cervix, and prostate [61,62,63,64,65]. It is important to note that many of these findings are based on histogram analytics of ADC values, suggesting value in an increased role for radiomics, and once again, the importance of voxelization. When it comes to differentiation between treatment effect and true progression with DWI, histogram analysis appears to render better results than whole lesion average values. However, valuable spatial information is lost when using this approach. Recent studies in gliomas have shown that decreased ADC does not equal tumor progression because coagulative necrosis in the central necrotic component of radiation necrosis can result in marked diffusion restriction. Therefore, the location of the low ADC values is key. Reduced ADC in the central necrosis suggests coagulative necrosis in the context of radiation necrosis whereas reduced ADC in the solid or enhancing lesion components suggests hyper-cellularity associated to recurrent tumor [24].

The use of DWI and ADC to distinguish pseudoprogression has also attracted interest. This has been amplified by the growth of targeted therapies and immunotherapies such as anti-PD1 and anti-CTLA-4 agents. Higher or intermediate ADC has been shown to correlate with pseudoprogression in various studies [66,67,68,69] whereas lower ADC has been shown to be precede new tumor growth [70]. However, further research is required to establish a consensus and these findings remain suggestive.

There has also been a diversification and expansion in the number of MRI systems, with the MRIdian System™ (ViewRay™, Cleveland, OH, USA) and the Unity system (Elekta AB, Stockholm, Sweden), being of particular note to radiation oncology through their on-board imaging integration. DWI during the course treatment is now feasible at an expanding number of radiation treatment centers [71], thus rapidly increasing opportunities for the use of imaging biomarkers to assess treatment response [72]. However, those wishing to take advantage of these opportunities should do so only after careful consideration of the literature, preferably including that associated with their particular imaging systems, consultation with medical physics specialists, and after the design and implementation of an appropriate quality assurance program to ensure the accuracy, repeatability, reproducibility of measures (see Section 5).

#### 3.2.2. MR Spectroscopy

Proton (1H)-magnetic resonance spectroscopy (MRS) is an established molecular profiling technique used in many research centers worldwide for pathological assessments and provides information regarding the presence and concentration of various metabolites. The large voxel size of MRS, however, is a limitation when it comes to metabolic profiling of highly heterogeneous diseased tissues such as tumorous tissue. This technique has been commonly used for differentiation between radiation necrosis and recurrent tumor [73,74,75]. In recurrent tumor, choline elevation is often observed whereas in radiation change, N-acetylaspartate, choline and creatine peaks will all be low. In current clinical practice, perfusion imaging is more commonly used than MR spectroscopy when evaluating tumor response [76,77].

Complementary to 1H-MRS for metabolic imaging is Chemical Exchange Saturation Transfer (CEST)-magnetic resonance imaging (MRI). CEST is an imaging technique, which exploits chemical exchange of labile protons of a particular (endogenous or exogenous) CEST agent with water to detect the former indirectly with high sensitivity through the water signal. Specifically, amide proton transfer (APT) CEST, which relies on the saturation of the amide protons including those within peptide bonds, shows signals in brain tumors related to increased levels of proteins and peptides [78]. Specific to glioma, CEST studies at both 3T and 7T have suggested the potential to predict response to therapy and offer unique signal characteristics and sensitivity to pH. A recent study by Chan et al. (2020) suggested a pulsed CEST/MT approach to be feasible on a 1.5T linac-integrated MRI and allow for distinguishing early from late progression GBM cohorts [79].

#### 3.2.3. PET Imaging

Tumor hypoxia is a key mechanism in disease progression and early identification of regions at risk for recurrence and prognostic-based classification of patients is necessary to formulate personalised therapeutic strategies. Leimgruber et al. [80] developed an image-based algorithm to spatially map areas of aerobic and anaerobic glycolysis (glyoxia) using 18F-FDG and 18F-FMISO PET in glioblastoma. They found that glyoxia-generated images were consistent with disease relapse topology and showed more prominent variation than hypoxia-based information alone. This highlights that spatial mapping of aerobic and anaerobic glycolysis allows unique information on tumor metabolism and hypoxia, thus providing a greater understanding of tumor biology and potential response to therapy. Key to acquiring these parametric maps is therefore the ability for biological imaging techniques to provide voxelwise information.

Among the most extensively studied imaging modalities for better understanding how primary brain tumor biology is reflected in the imaging phenotype are O-(2-[18F] fluoroethyl)-L-tyrosine (FET)-PET and DSC perfusion imaging. Using FET as a tracer, PET can visualise the amino acid uptake in gliomas and thus metabolically active tumor cells [81]. MR-based DSC perfusion provides evidence of neoangionesis and can help predict patient survival and response to anti-angiogenic therapy (bevacizumab) [82] but is not often routinely available for radiotherapy treatment planning or follow-up imaging. 

A study of brain metastases treated with SRS investigated the correlation of 18F-Fluorocholine uptake in surgical samples with pathologic evidence of recurrent tumor [83]. Strong correlation was observed between surgical SUVmax and PET imaging. However, 18F-fluorocholine count data was not only driven by viable tumor but also by degree of inflammation and reactive gliosis. 

More recently, amide proton-transfer-weighted (APTw) imaging is a MRI technique that semi-quantitatively reflects the concentration of endogenous proteins and peptides [84]. In newly diagnosed gliomas, evidence was found for a relevant overlap of tumor areas defined by established cut-offs for APTw and FET, both in contrast enhancing tumor and FLAIR-hyperintense tumor. Here too, synergistic use of multi-modality analysis found a correlation between cellularity for both imaging modalities that helped with tumor grading and differentiation between progression and pseudo-progression. These types of information rich datasets can act as a strong basis for training machine learning classifiers which are able to integrate the multimodal data input. 

## 4. Multi-Parametric Correlates of Tumor Microenvironment (TME)

Similar to the benefits of a unified pharmacokinetic modeling approach for DCE imaging [38], is the concept of an integrated TME feature model using multi-modal analysis to provide a better description of the tumor functional status. A study by Li et al. (2019) investigated an unsupervised patient clustering technique and showed that selected histogram features from perfusion and diffusion MRI can offer incremental prognostic values over clinical variables in glioblastoma patients [85]. 

One disadvantage of a voxel-wise approach such as the above, is that accurate co-registration of the data is necessary. Fortunately, several algorithms and software exist for performing co-registration and MRI manufacturers have developed automatic MRI slice positioning protocols (e.g., AutoAlign) that enable the precise and consistent alignment of scans among different individuals and repeated imaging of the same individual [86]. Voxel resampling is equally important and needs to be done in 3D prior to processing the data using interpolations of the reference and target radiological images without a loss of information. 

The construction of TME feature model can be broadly classified into supervised or unsupervised algorithms. Unsupervised algorithms use the inherent data heterogeneity within a given dataset to automatically determine distinct features between different tissue classes, e.g., malignant vs. healthy tissue. Supervised algorithms, in contrast, “learn” to distinguish tissue classes using training sets that are representative of data acquired from patients with the same disease/tumor type in which the state of tumor stage/response is known, e.g., measured with histopathology or on follow-up imaging. In supervised learning algorithms, the outcome states are *a priori* determined, and a training dataset is used to develop a model that minimizes misclassification rates in the training data. With supervised algorithms, one needs to be careful not to “overfit” on the training data in order for the model to be applicable to other datasets. 

Perkuhn et al. (2018) [87] investigated preoperative MRI scans (T1, T2, FLAIR, and contrast-enhanced [CE] T1) of 64 patients with an initial diagnosis of primary glioblastoma, which were acquired in 15 institutions with varying protocols. All images underwent preprocessing (co-registration, skull stripping, resampling to isotropic resolution, normalization) and were fed into an independently trained deep learning model based on a multilayer, multiscale convolutional neural network for detection and segmentation of tumor compartments. Automatic segmentation results for the whole tumor, necrosis, and contrast enhanced tumor were compared with manual segmentations. The proposed approach for automatic segmentation of glioblastoma proved to be robust and showed on all tumor compartments a high automatic detection rate and a high accuracy, comparable to interrater variability. Automatic segmentation of brain metastases was also investigated using deep learning CNN based on GoogLeNet architecture and found to produce excellent detection and segmentation accuracy compared to manual segmentations by 2 experienced neuroradiologists [88]. A preliminary multi-parametric radiomics approach in small data set of 24 patients with brain metastases, was further able to distinguish true progression from pseudoprogression [89] and demonstrated that this approach could provide higher classification accuracy than single parameter radiomics features. 

## 5. Reliability/Variability of Quantitative Imaging Biomarkers

Imaging biomarkers are important in oncology as they non-invasively provide information about the tumor and can be used for treatment response monitoring [72]. Quantitative imaging biomarkers (QIBs) are of particular interest because they provide quantitative information about tissue characteristics [3]. Ideally, they facilitate the comparison across different vendors and centers. However, differences in system hardware, acquisition parameters, and image analysis techniques introduce variability of QIB values [56,90]. It is critical to understand these differences and to test and validate QIBs before they can be incorporated in clinical trials [90,91,92]. 

The diversification of systems exacerbates a pre-existing barrier to the increased exploitation of, in particular, DWI related biomarkers—a lack of convincing repeatability and reproducibility studies [93]. Sequence design can be limited by manufacturer constraints, preventing standardization across machines. DWI is known to have large geometric distortions which are a barrier to their use in targeting radiotherapy, but also lead to uncertainties in measured ADC and IVIM across the imaging space. 

The statistical requirements to reliably differentiate between measurement uncertainty and true biomarker change in response to treatment are described in more detail in a QIBA recommendation paper for DCE MRI and DWI measurements [93]. 

Careful consideration of sequence design and a comprehensive quality assurance (QA) program is an essential part of any program hoping to obtain imaging biomarkers. Testing with phantoms is important and will provide assurance that the measures obtained are reliable [94]. Repeatability measures, such as test-retest studies, should also be conducted wherever feasible. Publications from QIBA and consortia relevant to the imaging unit being used [71,95,96] should be sought and be used as a foundation for sequence and quality assurance program design.

The introduction of hybrid systems, which integrate an MRI with a linear accelerator (MR-linac), presents a unique opportunity for QIB studies. MRI-guided treatments enable QIBs to be acquired daily, which is practically not feasible on diagnostic MRI systems and will provide valuable longitudinal information. However, to maximize the power of these QIB studies, it is critical to harmonize the acquisition protocols across centers [71].

Translation of diagnostic investigations to MR-guided delivery systems requires machine characteristics to be considered. For example, when designing protocols for ADC measurement, b-values should be chosen with care, consulting the literature for that machine [71], and consideration given to the characterization of ADC accuracy across the imaging space with subsequent ROIs chosen accordingly. Factors such as linear-accelerator gantry angle on hybrid MRI-linear accelerators add additional uncertainty [71,95,96] which require quantification.

In summary, a quality assurance program with support from medical physics is essential, protocol and quality assurance programs should be bespoke for an individual center and technology, test-retest measurements should be obtained wherever possible, and the measurement uncertainty should be properly characterized across all conditions and the imaging space. 

## 6. Current vs. Next Steps

Advanced imaging techniques based on QIB are promising in the yet gray areas of response assessment in neuro-oncology, but its use as objective clinical and clinical trial endpoints still needs accuracy and reproducibility validation. Complimentary to the need for improved evaluation metrics of treatment response, is the requirement of improved trial design to determine the role of QIB and allow the evaluation of its reliability and prognostic ability.

Imaging interpretation requires profound knowledge of the tumor biology after operation, radiation, and chemotherapy, since tumors respond differently to different therapies. To date manual or semi-automated quantification of tumor volume and response by experts using fancy tools is variable across platforms, is time-consuming and expensive. This all leads to neuro-radiologists expending extra time and energy during the response assessment effort. 

Radiological findings play a critical role in the assessment and management of patients with brain tumors, and it is not rare that progression is missed on imaging, causing irreparable consequences to patients’ QoL. This is one of the reasons why finding the optimal timing of imaging is important. Using RANO or iRANO/iRECIST to describe changes when assessing brain tumors characteristics (T2/flair or T1 with contrast) can be challenging and time consuming. One example is the iRECIST protocol, which addresses specific criteria to assess tumor response in imaging after immunotherapy since the therapy itself can cause apparent tumor enhancement due to inflammation—and, because of that, should the patient present with worsening on imaging within 6 months of treatment and remains clinically stable, they should continue the course of treatment [97,98]. 

Artificial intelligence (AI) has shown to be a promising field in neuro-oncology [99]. The development of automated algorithms that use deep learning to perform imaging readings in neural conditions has shown positive results, with a range of benefits such as decreasing inter-observer variability or maximizing time efficiency in manual tumor segmentation, and its use has been well described in other areas of oncology. Automated readings of imaging can be used to improve and facilitate reliable use of endpoints, reducing time to assess tumors and physician burden associated with manual tumor segmentation. AI tools can also be integrated into clinical workflow, providing reproducible results and efficiency to patient assessment. One further use of AI is in management of disease, since rapid automated segmentation allows for strict comparison of tumor volume and sizes in patients undergoing therapy [99]. 

However, in order for AI to be used in the clinical setting, institutions must provide infrastructure for efficient image processing and in-site AI data mining, which currently poses as the major barrier to its wide implementation. Furthermore, it is important that research and multicenter clinical trials are performed to assess the role of AI, and validate the use of this technique as a reliable and precise manner to assess patients with brain tumors, as well as develop more precise techniques that account for anatomic variability and dataset differences [99]. Future work will prove that volume is not the only feature that can be extracted for response assessment and that assessing intensity, texture and hidden pattern will be of great use improve prediction of disease progression in oncology. 

Following guidelines outlined in previous publications, this review offers a broad overview of the subject, which can be useful as an educational tool for this innovative topic. It is worth noting that the main drawback of narrative reviews is their unsystematic method of article selection which could result in a bias in the overall analysis of findings. However, due to paucity of information on the topic, the authors decided to proceed with this type of review regardless, in order to contribute to the literature by addressing such topics and encouraging discussion.

## 7. Conclusions

Functional and metabolic imaging approaches have evolved significantly over the last decade and the voxelization of these non-invasive, repeatable approaches is very well suited to describe the heterogeneous nature of the tumor microenvironment. Here we discussed the most widely developed biological imaging techniques reported in the literature in the context of radiation and immunotherapy to primary and metastatic brain tumors. Some, such as DCE and DSC MRI are different approaches to obtaining a similar metric, e.g., perfusion. Others, such as DWI and PET hypoxia imaging are more complementary in nature; but all aim to aid in providing the fullest description possible of a TME model that has a high correlation/prognostic ability to evaluate a tumor’s staging and its response to treatment. There is no doubt that other techniques will soon start to play a stronger role in contributing to this model: MR spectroscopy, arterial spin-labeling MRI and FLT-PET to just name a few.

The concept of what constitutes a reliable clinical endpoint is an important consideration in this new realm. Complimentary to the need for improved evaluation metrics (RANO/RECIST) of treatment response, is the requirement of improved trial design to determine the role of QIB and allow the evaluation of its reliability and prognostic ability. The community, through efforts of international agencies such as e.g., QIBA and QIN, as well as TCIA and MRL consortia are supportive of the efforts to establish data and clinical trials geared towards validating the QIB against adapted endpoints. The latter have matured beyond progression free or overall survival to also include QoL and neuro-cognition metrics.

The technological advances in imaging acquisition and analysis techniques have matured to a strong enough foundation for the end goal of prognostic QIB to be in sight. The role of deep learning and AI in developing the second phase of this journey has been shown, but requires large, well curated, validated data sets to be further developed. This can only be achieved through collaboration and standardization, supported by automation and innovation. 

## Data Availability

No new data were created or analyzed in this study. Data sharing is not applicable to this article.
